# Supplementary material for: Mask-inspired moisture-transmitting and durable thermochromic perovskite smart windows
Source: Nat Commun. 2024 Jan 30;15:876. doi: 10.1038/s41467-024-45047-y (PMC10827790; doi:10.1038/s41467-024-45047-y)
Supplement: Supplementary file 1 — Supplementary Information [file 41467_2024_45047_MOESM1_ESM.pdf]

# Supplementary Information

## Mask-Inspired Moisture-transmitting and Durable Thermochromic Perovskite Smart Windows

Sai LIU, Yang LI, Ying WANG, Yuwei DU, Kin Man YU, Hin Lap YIP, Alex K.Y. JEN, Baoling HUANG*, Chi Yan TSO*

*Corresponding author. Email: chiytso@cityu.edu.hk (CYT), mebhuang@ust.hk (BLH)

**This PDF file includes:**

Supplementary Figure 1 to 24
Supplementary Table 1 to 3
Supplementary Text 1 to 3

**Other supplementary information for this manuscript includes the following:**

Supplementary Movie 1 to 4

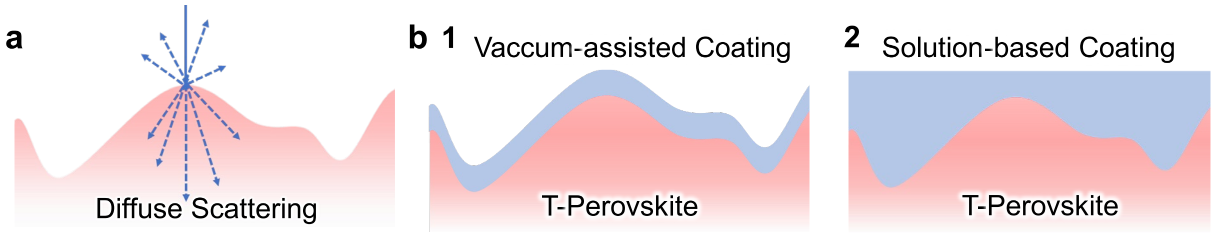


**Supplementary Figure 1.** (a) Light transmission through a rough surface. (b) Schematics of the high-vacuum deposition and solution-based coatings. When coated on rough surfaces, the solution-processed coating can fill the roughness and form a smooth surface.

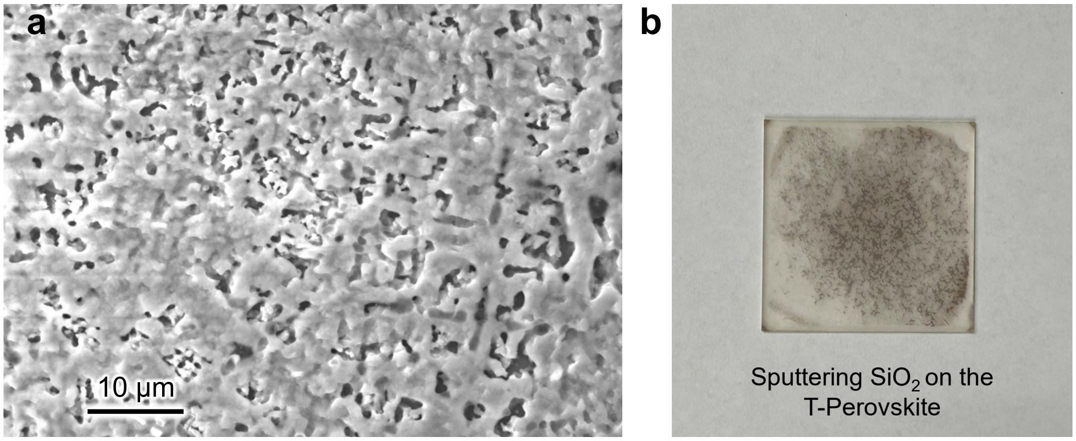


**Supplementary Figure 2.** (a) SEM image of sputtering $SiO_2$ on the T-Perovskite. The rough surface implies that sputtering method is unable to improve the surface morphology. (b) Photos of the T-Perovskite after the magnetron sputtering. These fadeless brown spots on its surface indicate that the high-energy plasma during the sputtering process can damage the T-Perovskite.

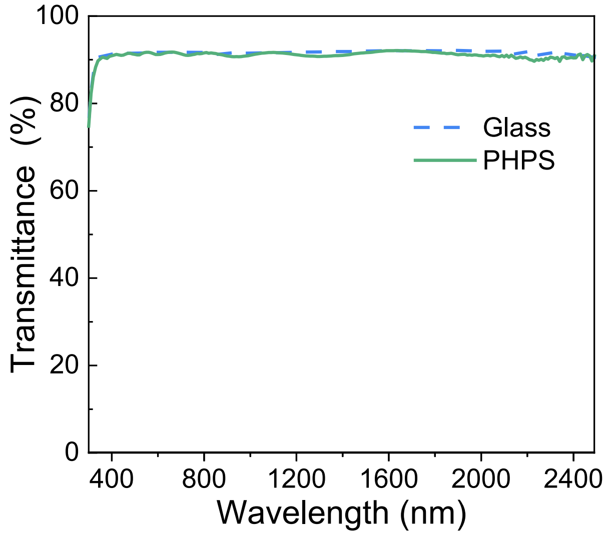


**Supplementary Figure 3.** Transmittance spectra of the glass and PHPS-coated glass, showing the high transparency of the PHPS coating. The thickness of the PHPS coating was ~1.8 μm. Source data are provided as a Source Data file.

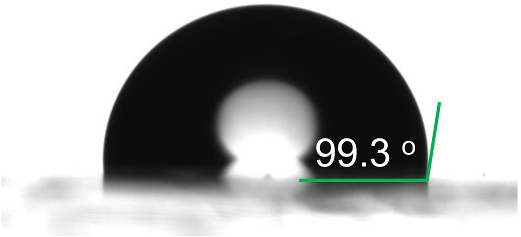

**Supplementary Figure 4.** Contact angle of PHPS coated on glass.

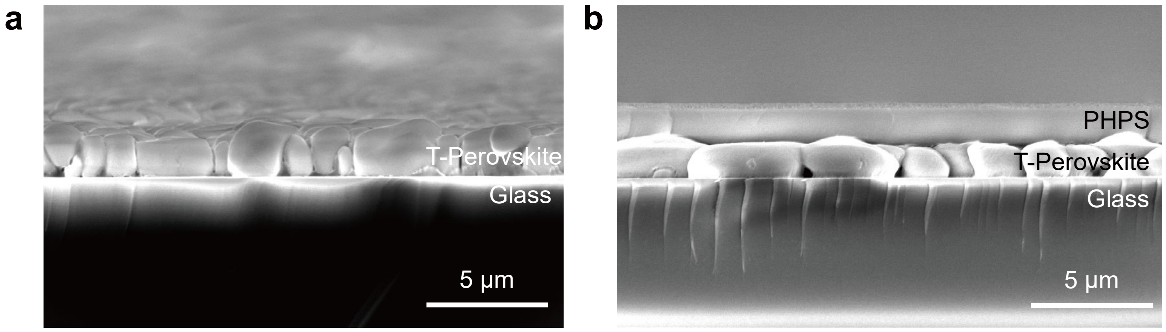


**Supplementary Figure 5.** Cross-sectional SEM images of the (a) T-Perovskite coated on glass and (b) PHPS coated on the T-Perovskite.

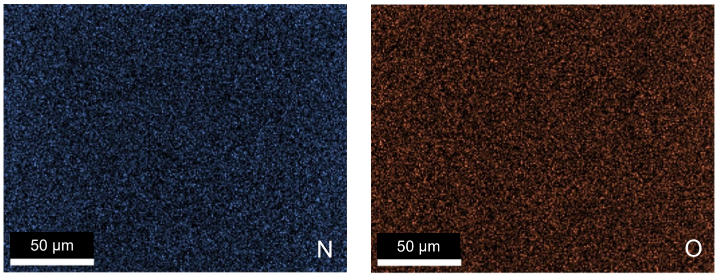


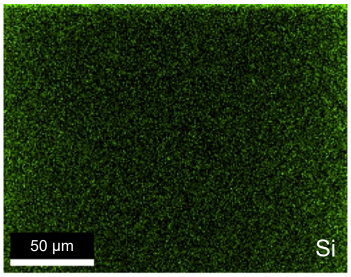


| Element | Weight % | Atomic % |
|---|---|---|
| N K | 36.9 | 43.9 |
| O K | 41.6 | 43.3 |
| Si K | 21.5 | 12.7 |

**Supplementary Figure 6.** EDS analysis of the PHPS film.

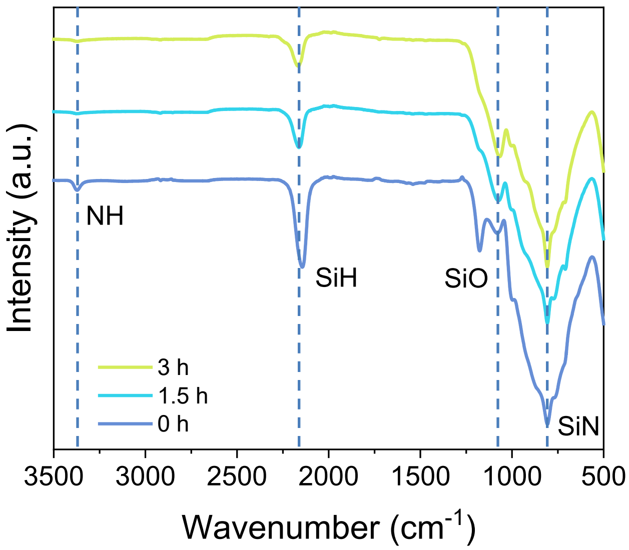


**Supplementary Figure 7.** FTIR spectra of the PHPS in the curing process. Source data are provided as a Source Data file.

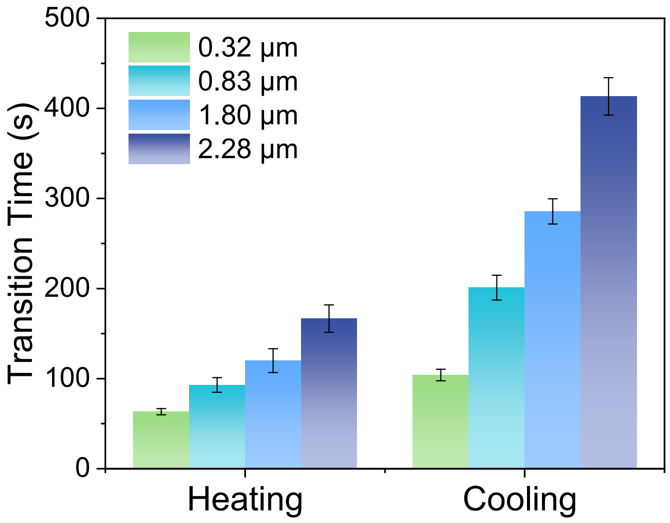


**Supplementary Figure 8.** The transition (color switch) time of T-Perovskite upon heating cooling with different thicknesses of the PHPS coating. (the error bars represent the standard deviations from three parallel measurements). Source data are provided as a Source Data file.

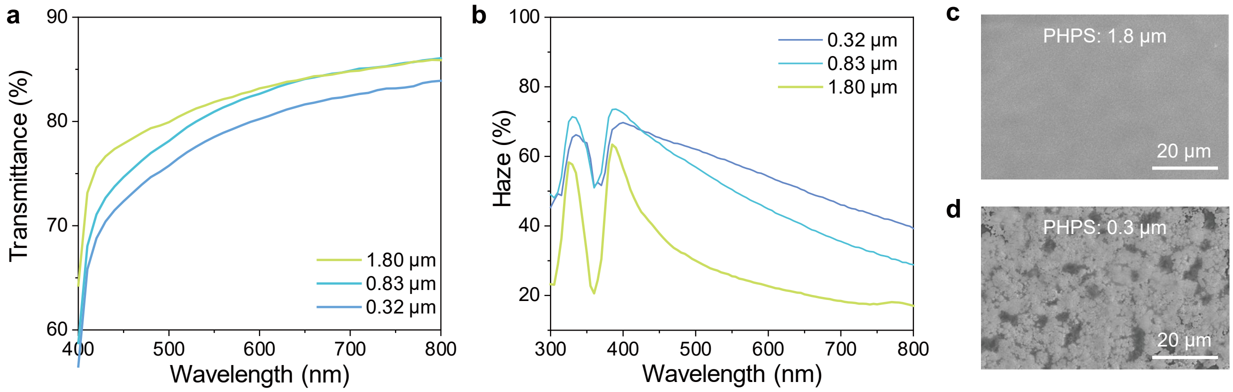


**Supplementary Figure 9.** (a) The transmittance variation by coating different thicknesses of PHPS on the T-Perovskite. (b) The haze variation by coating different thicknesses of PHPS on the T-Perovskite. (c) SEM image of 1.8 µm PHPS-coated T-Perovskite. The flat surface indicates uniform coverage. (d) SEM image of 0.3 µm PHPS-coated T-Perovskite. The rough surface indicates poor coverage. Source data are provided as a Source Data file.

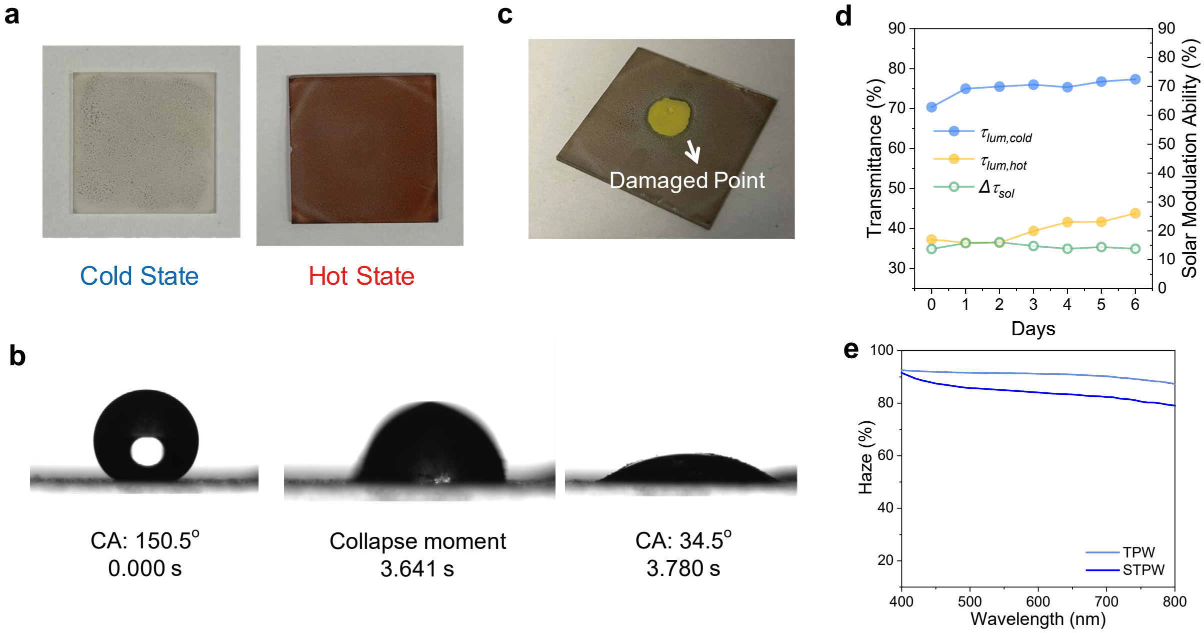


**Supplementary Figure 10.** Hydrophobic $SiO_2$ nanoparticles spray-coated T-Perovskite window (STPW) without the PHPS layer. (a) The STPW at cold and hot states. The black surface of the T-Perovskite at the cold state is attributed to the damage from the ethanol used for $SiO_2$ nanoparticles dispersion. (b) Contact angle change of the STPW in a short 3.78 s. (c) The STPW was damaged by the water droplet. (d) Durability test for the STPW in the ambient environmental condition (23 °C, RH = 60%). (e) Haze comparison of STPW and bare thermochromic perovskite window (TPW). Source data are provided as a Source Data file.

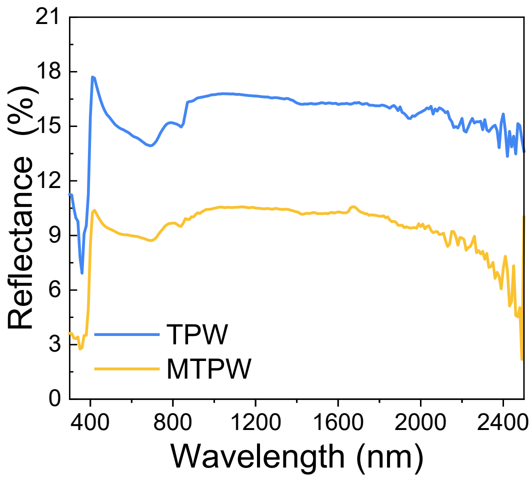


**Supplementary Figure 11.** The reflectance profiles of TPW and MTPW. Source data are provided as a Source Data file.

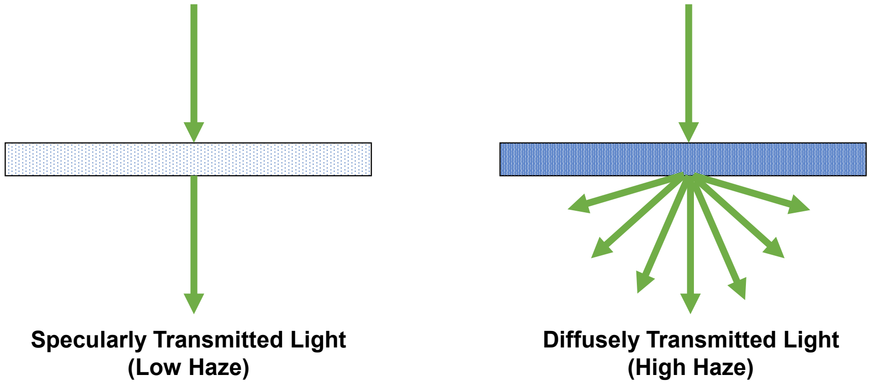


**Supplementary Figure 12.** Schematics of specularly transmitted light and diffusely transmitted light.

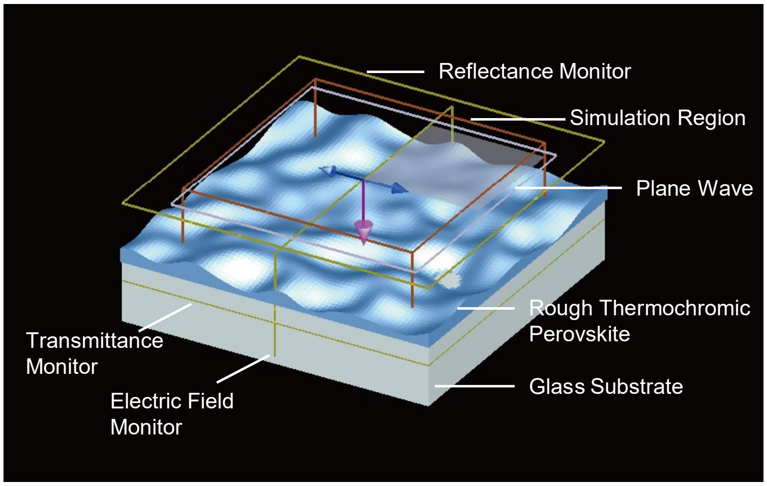


**Supplementary Figure 13.** Schematic of the FDTD model to simulate light transmission, reflection and propagation directions.

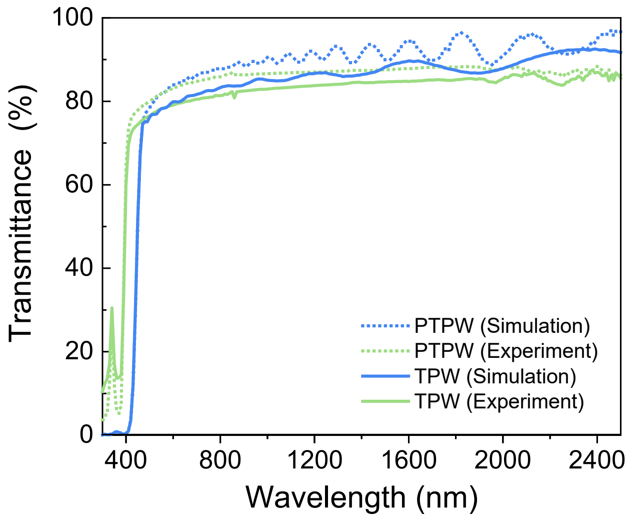


**Supplementary Figure 14.** Transmittance spectra from FDTD simulations and experiments to verify the FDTD model. This figure also shows that the transmittance of the PTPW is higher than that of the pristine TPW in both the simulated and experimental results. Source data are provided as a Source Data file.

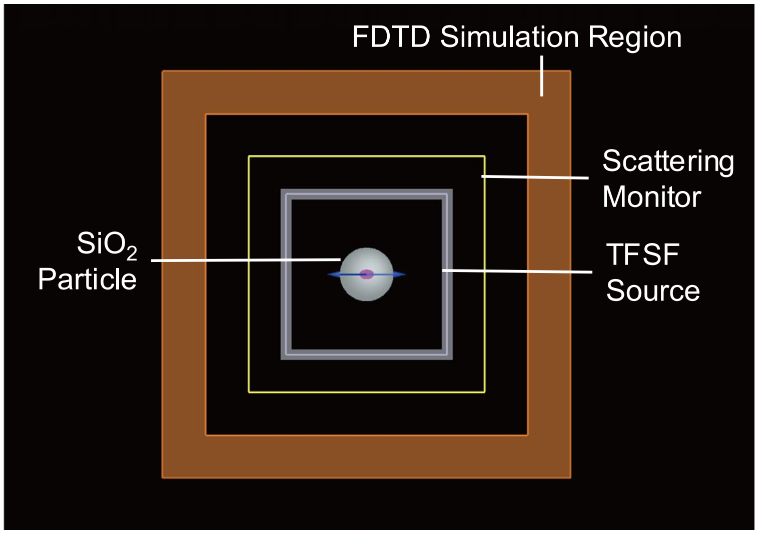


**Supplementary Figure 15.** FDTD simulation model to calculate the scattering efficiency of a $SiO_2$ nanoparticle.

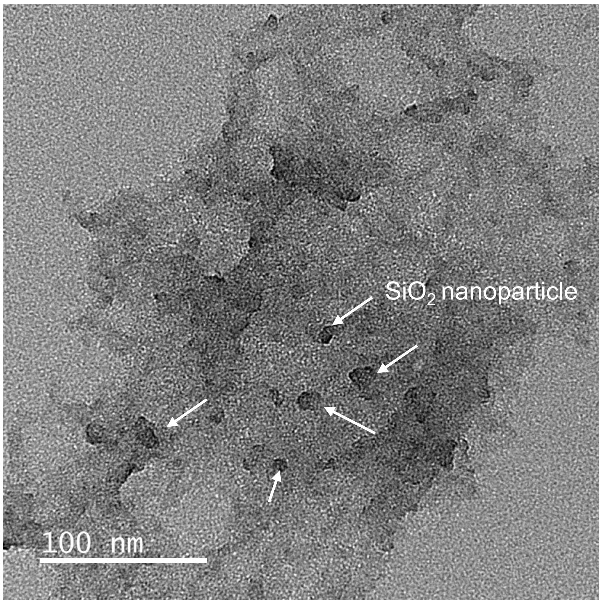


**Supplementary Figure 16.** TEM image of $SiO_2$ nanoparticles on the top layer of the MTPW.

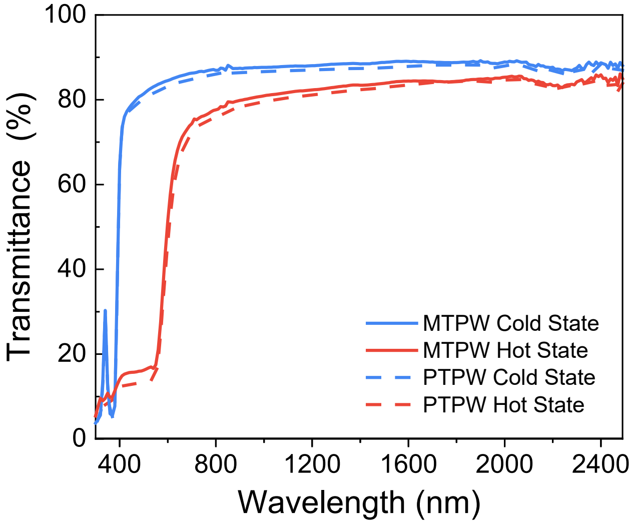


**Supplementary Figure 17.** Comparison of the transmittance spectra of the PTPW and MTPW to verify that the $SiO_2$ nanoparticles do not impair the transparency. Source data are provided as a Source Data file.

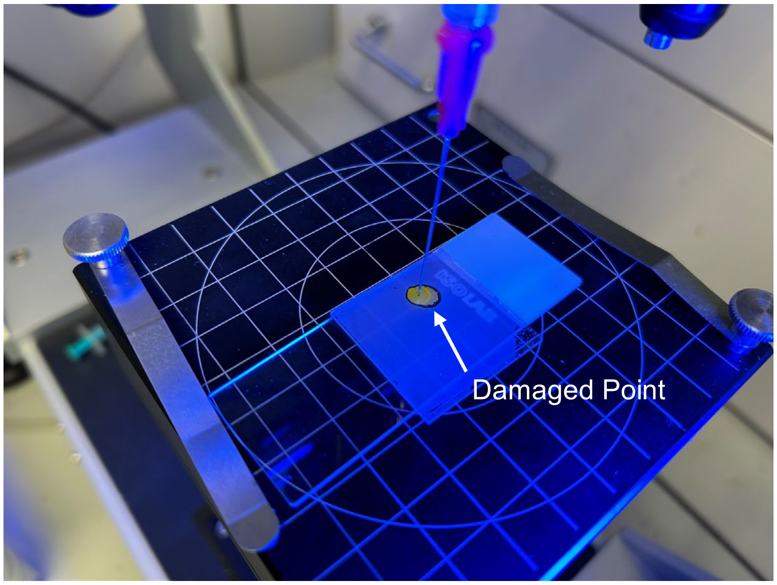


**Supplementary Figure 18.** Image of dripping water on the pristine TPW, showing that the surface was rapidly damaged.

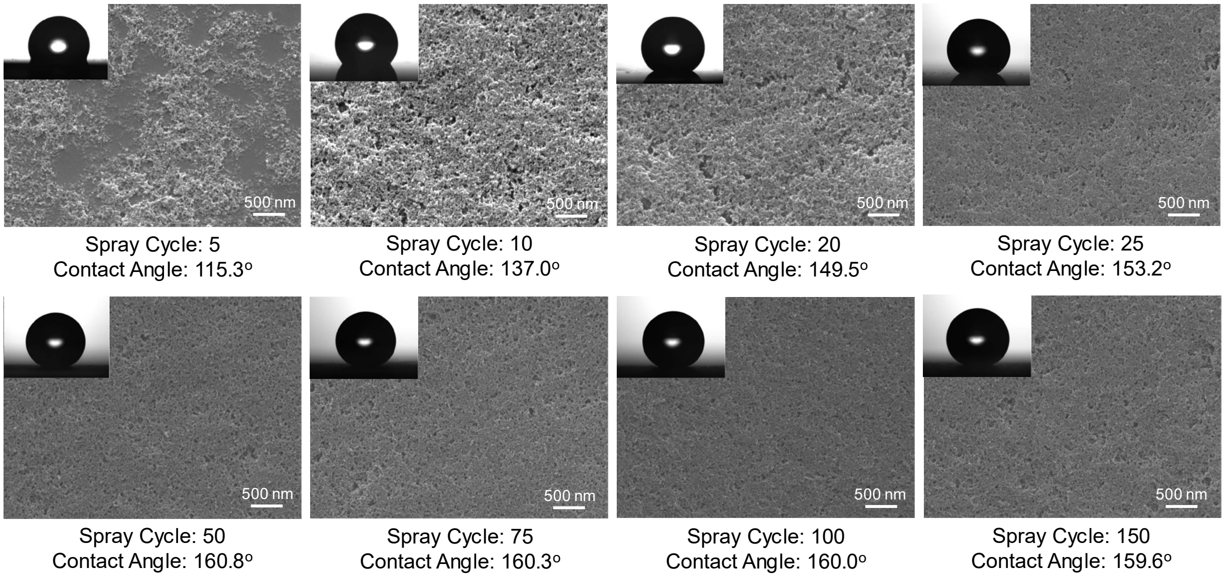


**Supplementary Figure 19.** SEM top-view images showing the surface morphology obtained by spraying $SiO_2$ nanoparticles for different numbers of cycles and their corresponding CAs.

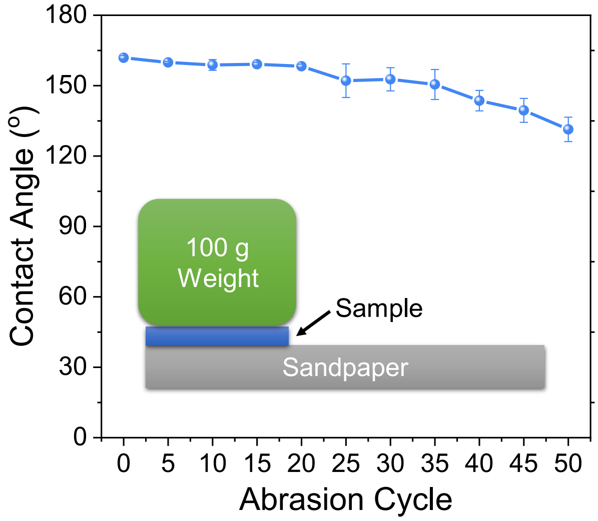


**Supplementary Figure 20.** Water contact angle of $SiO_2$ nanoparticles coating after sandpaper abrasion. The error bars represent the standard deviations from three parallel measurements. Source data are provided as a Source Data file.

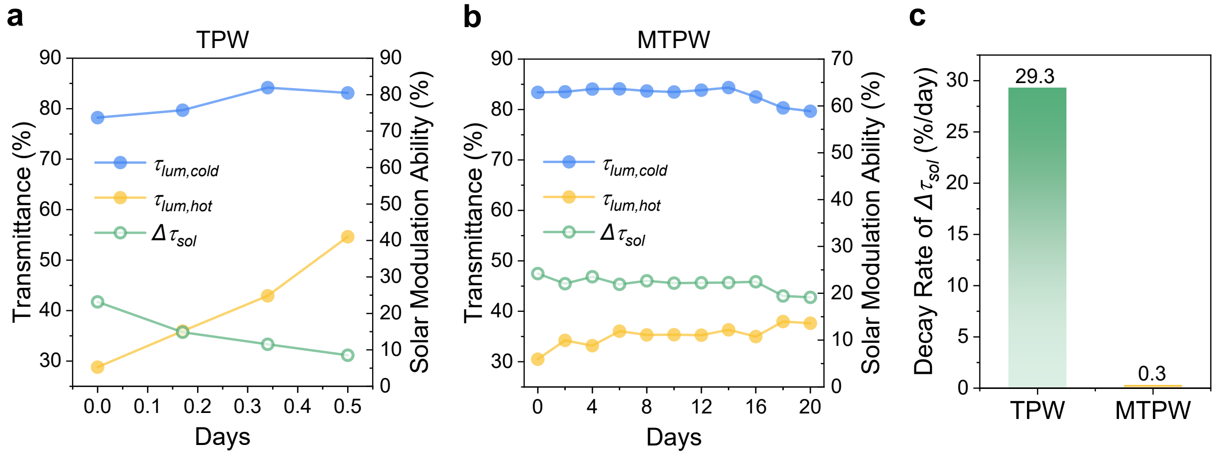


**Supplementary Figure 21.** (a and b) Optical performance ($\tau_{lum,hot}$, $\tau_{lum,cold}$ and $\Delta\tau_{sol}$) of the pristine TPW and the MTPW in the hot and humid environment. (c) Decay rate of $\Delta\tau_{sol}$ for the TPW and MTPW in the hot and humid environment. Source data are provided as a Source Data file.

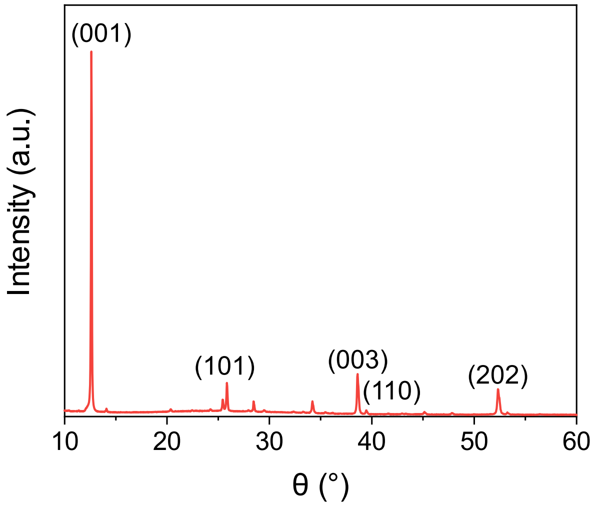


**Supplementary Figure 22.** XRD pattern of a pristine T-Perovskite thin film immersed in water. The main observed peaks are in accordance with those of $PbI_2$. Source data are provided as a Source Data file.

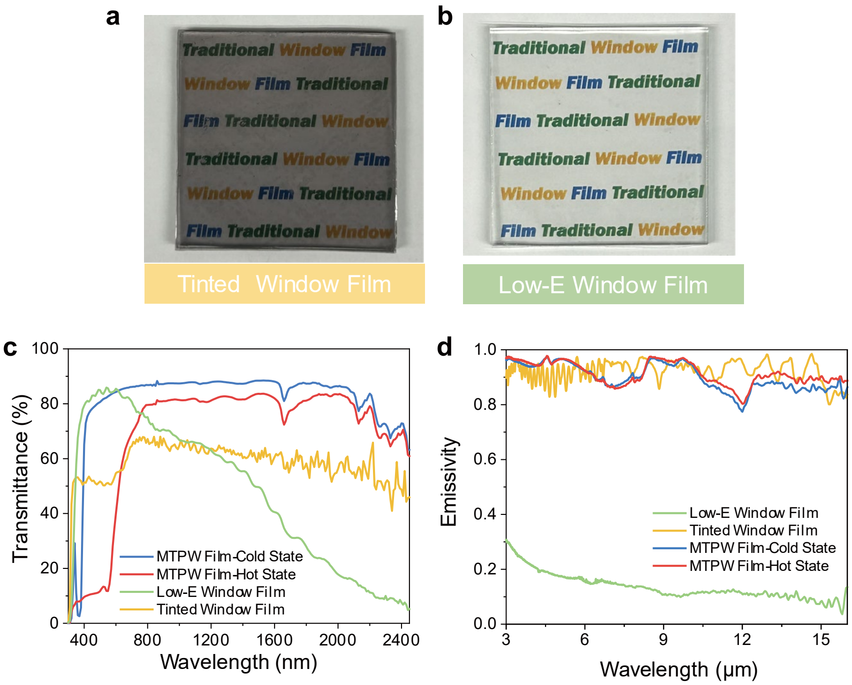


**Supplementary Figure 23.** Photographs of (a) a commercial energy-saving tinted window film and (b) a commercial energy-saving Low-E window film (c) Transmittance spectra of the commercial window films and MTPW film. (d) Emissivity spectra of the commercial window films and MTPW film. Source data are provided as a Source Data file.

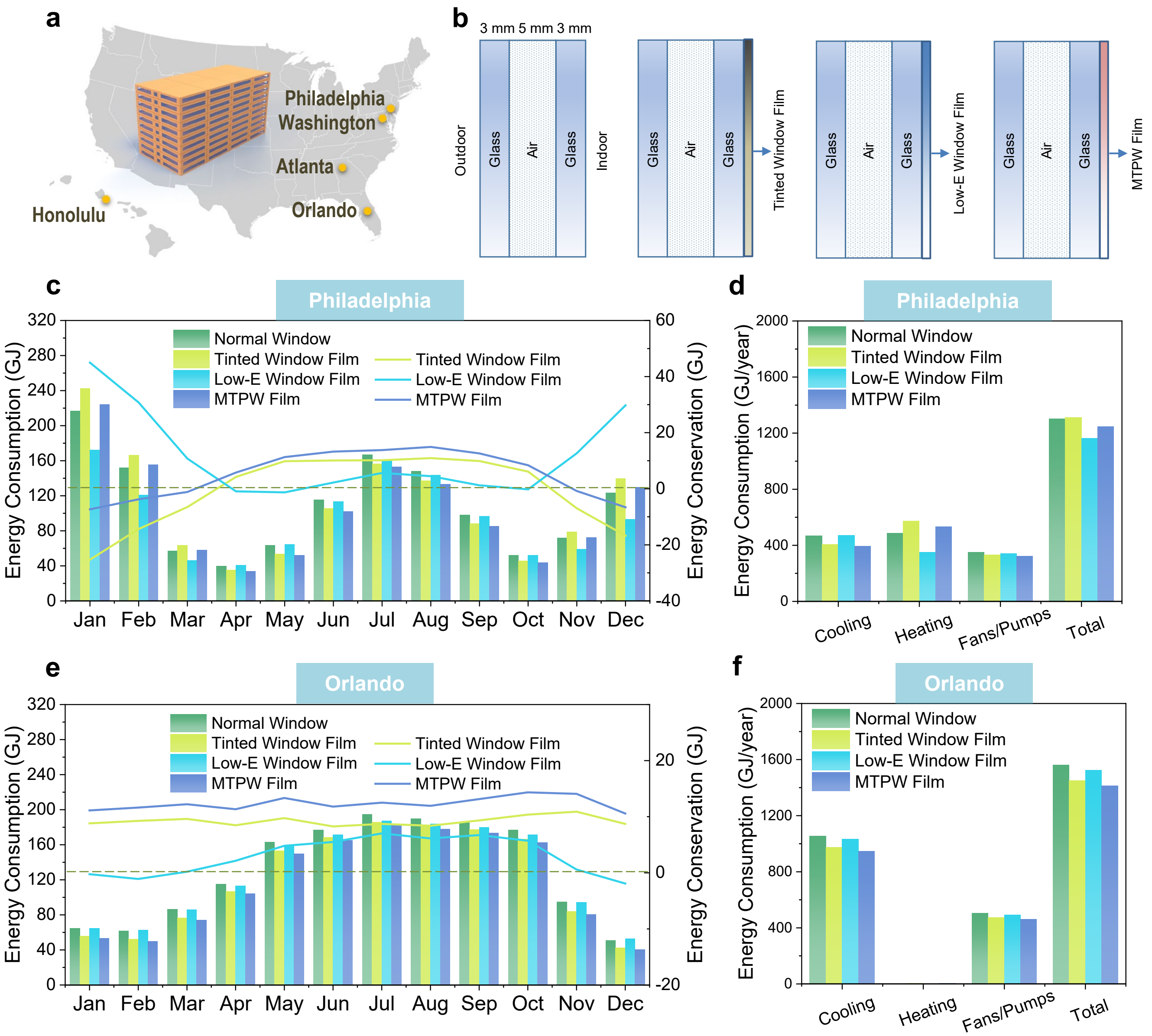


**Supplementary Figure 24.** (a) Locations of Philadelphia, Washington, Atlanta, Orlando and Honolulu as well as the building model used in the EnergyPlus simulation. (b) Window structures (normal window, tinted window film, Low-E window film pasted on the window, and MTPW window film pasted on the window) in the EnergyPlus simulation. (c) Monthly energy consumption and savings in Philadelphia using different windows. (d) Energy consumption by types in Philadelphia using different windows. (e) Monthly energy consumption and savings in Orlando using different windows. (f) Energy consumption by types in Orlando using different windows. Source data are provided as a Source Data file.

**Supplementary Table 1.** Information of the building used in the EnergyPlus simulation.

| | |
|---|---|
| Building type | Apartment |
| Number of Floors | 10 |
| Total Floor Area | 46.3 m × 16.9 m |
| Average Window-to-Wall Ratio | 30% |
| HVAC System | Water source heat pumps |
| Temperature Set Point for HVAC Control | Below 21.1 °C for heating/Above 23.8 °C for cooling |

**Supplementary Table 2.** Optical information of the windows used in the EnergyPlus simulation.

| | Normal Window | Commercial Window Film Pasted on the Window | Low-E Film Pasted on the Window | MTPW Film Pasted on the Window | |
|---|---|---|---|---|---|
| States | - | - | | Cold State | Hot State |
| Solar Transmittance | 0.6205 | 0.4109 | 0.5279 | 0.5883 | 0.3536 |
| Solar Front Reflectance | 0.1933 | 0.1690 | 0.1760 | 0.1888 | 0.1788 |
| Solar Back Reflectance | 0.2276 | 0.1440 | 0.1701 | 0.2075 | 0.1428 |
| Visible Transmittance | 0.7222 | 0.4277 | 0.6971 | 0.6922 | 0.1934 |
| Visible Front Reflectance | 0.2309 | 0.2003 | 0.2115 | 0.2337 | 0.2161 |
| Visible Back Reflectance | 0.2357 | 0.1251 | 0.2007 | 0.2304 | 0.1934 |
| Emissivity | 0.8400 | 0.9396 | 0.1090 | 0.9056 | 0.9207 |

**Supplementary Table 3.** Climate information for cities in America used in the EnergyPlus simulation.

| City (from north to south) | Climate Types | Average Temperature (1991-2020) |
| --- | --- | --- |
| Philadelphia | **Continental:** cold winters and hot summers | Mean: 1.3–25.6 °C<br>Max: 31.2 °C<br>Min: -3.3 °C |
| Washington (D.C.) | **Semicontinental:** cold winters and hot, muggy summers. | Mean: 3–27.2 °C<br>Max: 32 °C<br>Min: -1.1 °C |
| Atlanta | **Humid subtropical:** mild and rainy winters and hot, muggy summers. | Mean: 7.1–27.2 °C<br>Max: 32.3 °C<br>Min: 2 °C |
| Orlando | **Subtropical:** very mild winters and hot, humid summers. | Mean: 15.9–28.1 °C<br>Max: 33.3 °C<br>Min: 9.7 °C |
| Honolulu | **Tropical:** a hot season from June to October and a relatively cool season from December to March. | Mean: 23.1–27.9 °C<br>Max: 31.6 °C<br>Min: 19.3 °C |

**Supplementary Text 1:**

Light propagation through a rough surface:

Along the plane wave propagation path, different parts of the wavefront encounter a rough surface at different heights. Therefore, the scattered components have a phase difference. With respect to the components transmitted from point A of the rough surface and the flat surface at the mean height $[h]$, the phase difference is:

$$\Delta\varphi = k\Delta h(n_1\, cos\alpha_i - n_2\, cos\alpha_t),\ (1)$$

$$k = \frac{2\pi}{\lambda},\ (2)$$

$$\Delta h = h_A - [h],\ (3)$$

where $k$ is the wavenumber in vacuum, $\Delta h$ is the height variation around the mean value of the rough surface height $[h]$, and $n$ is the refractive index of the media. According to the Rayleigh roughness criteria, the standard deviation of the phase difference should be less than π/2 to ensure constructive interference (i.e., spectacular transmission). In this case, the surface can be considered as a smooth surface. To meet the Rayleigh roughness criteria, based on equations (1)-(3), the following must hold:

$$\sqrt{\frac{\sum_{A=1}^{N}(h_A-[h])^2}{N}} = RMS < \frac{\lambda}{4(n_1\, cos\alpha_i - n_2\, cos\alpha_t)}.\ (4)$$

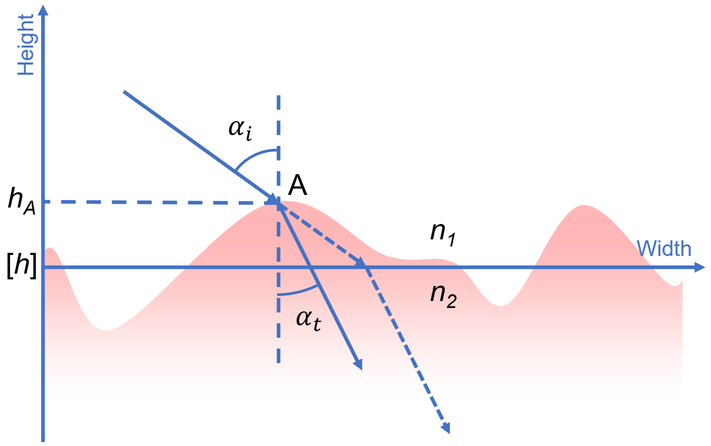

**Supplementary Text 2:**

EnergyPlus simulation analysis:

To compare the energy-saving performance, EnergyPlus modeling was conducted for a commercial building using a commercial tinted film, Low-E film and the MTPW film in five cities of the US (Supplementary Fig. S24A and B). The basic building information is listed in Supplementary Table S1, and the optical information of the normal glass window, the window with the tinted film, the Low-E film and the MTPW film used in the simulation was calculated via the WINDOW algorithm developed by the Lawrence Berkeley National Laboratory (Supplementary Table S2). The climate information of each city is listed in Supplementary Table S3.

Taking Philadelphia as an example, the heating energy consumption in buildings with tinted window films was much higher than that in buildings with normal windows, especially in winter from November to March (Supplementary Fig. S24C), which eventually offset the saved cooling energy in hot weather (Supplementary Fig. S24D). In contrast, enabled by the smart thermally responsive color switching ability, the MTPW maintained a high solar heat gain in cold weather but a low solar transmittance in hot weather. Therefore, the heating demand when using the MTPW film did not significantly change compared to that when using the normal window in winter, whereas the cooling demand dramatically decreased in the transition seasons and summer from April to October (Supplementary Fig. S24C), demonstrating the advantage of the smart optical regulation function. For the Low-E film with low IR emissivity, it has even better energy-saving potential than the MTPW film in winter from November to March. In the hot seasons from April to October, the Low-E film can still save energy due to the low NIR transmittance thus blocking part of solar radiation. But it does not perform remarkable energy-saving ability compared with the tinted film and MTPW film (Supplementary Fig. S24C and D). For hot areas (e.g., Orlando), the windows with the MTPW and tinted window film both showed better energy-saving ability than the normal window (Supplementary Fig. S24E and F), and the MTPW window exhibited better performance due to the lower solar transmittance at the hot state. However, Low-E window film suppresses the heat brought by solar radiation dissipating from the indoor side to the outdoor side, thus leading to low energy savings for cooling systems in subtropical and tropical areas.

**Supplementary Text 3:**

Parameter setting for the spray-coating machine:

The samples were placed on the vacuum stage of the spray machine, perpendicular to the spray nozzle. An ultrasonic spray system (UC 330, Siansonic Technology) equipped with a 45 kHz frequency nozzle (Z402, Siansonic Technology) was used to coat superhydrophobic $SiO_2$ nanoparticles. The concentration of the $SiO_2$ dispersion was 1 wt%. The details of the spray-coating parameters, including the flow rate, shaping air pressure, distance between the nozzle tip and substrate, nozzle speed, path width, number of layers, stage temperature, and ultrasonic power, are shown below. These parameters were chosen after multiple screenings and tuning of sample fabrication processes The thickness of each layer was optimized by layer-by-layer spray deposition. Layers were deposited in an alternating zigzag path with a 4 mm gap to cover the entire surface.

| Parameter: | Value: |
|---|---|
| Flow rate | 0.1 ml/min |
| Shaping air pressure | 0.015 MPa |
| Distance between nozzle and sample | 50.0 mm |
| Nozzle speed | 100 mm/s |
| Path width | 4 mm |
| Number of runs | 5/10/15/20/25/50/100/150 |
| Substrate temperature | 50 °C |
| Ultrasonic power | 3.0 W |
