# Supplementary material for: Mask-inspired moisture-transmitting and durable thermochromic perovskite smart windows
Source: Nat Commun. 2024 Jan 30;15:876. doi: 10.1038/s41467-024-45047-y (PMC10827790; doi:10.1038/s41467-024-45047-y)
Supplement: Supplementary file 3 — Description of Additional Supplementary Files [file 41467_2024_45047_MOESM3_ESM.pdf]

Description of Additional Supplementary Files

Supplementary Movie Legends:

Supplementary Movie 1. Reversible color switching of the MTPW between the cold and hot states to show the thermochromic effect.

Supplementary Movie 2. Light propagation process at the air/window interface of an ideally smooth TPW, a rough TPW and a PTPW in the FDTD simulation.

Supplementary Movie 3. Dripping water droplets test to demonstrate the superhydrophobicity of the MTPW.

Supplementary Movie 4. Water flushing test to examine the stability of the MTPW.
